# Identification and restoration of hydrological processes alteration during the fish spawning period

**DOI:** 10.1038/s41598-023-38441-x

**Published:** 2023-07-12

**Authors:** Yang Yu, Rui Zhao, Jiahe Zhang, Sen Du, Tianyu Zhou, Xingjia Fu, Shuoyun Jiang

**Affiliations:** 1 Faculty of Geosciences and Environmental Engineering, Southwest Jiaotong University, No. 999 Xi’an Road, Chengdu, 611756 China; 2 Sichuan Provincial Water Resources Department, River, and Lake Protection and Regulatory Affairs Center, No. 33 Qingjiang Road, Chengdu, 610072 China

**Keywords:** Environmental impact, Hydrology, Freshwater ecology

## Abstract

The hydrological processes play an important role in stimulating fish spawning behavior. Changes in the natural hydrological processes will alter the populations and distribution of fish, which may have a negative impact on the native aquatic organisms. The aim of this study is to identify the alteration of the water rising process during the fish spawning period and to construct an ecological flow optimization model to restore the water rising conditions for fish reproduction. The Mann–Kendall test and the sliding t-test were used to detect the mutation year of the mean daily flow data sets in the fish spawning period in each monitoring year. Then the data sets can be divided into pre-altered and post-altered periods. The water rising process was characterized by the water rising processes count, the duration, the daily flow increase rate, the date of the water rising process, and the initial water rising flow. The changes in hydrological processes in the middle reaches of the Yangtze River were investigated by comparing the post-altered and pre-altered characteristic parameters. Furthermore, we integrated the statistical values of the five characteristic parameters in pre-altered into an ecological flow optimization model to simulate the natural water rising processes for the spawning of the Four Major Chinese Carps (FMCC) and Chinese Sturgeon (CS). The analysis showed that after the hydrological mutation year, the duration and the initial water rising flow in the FMCC spawning season were increased, with hydrological alteration degrees of 63.10% and 70.16%, respectively; however, the daily flow increase rate was significantly decreased, with hydrological alteration of 86.50%. During the CS spawning season, the water rising processes count and the initial water rising flow were dramatically altered parameters, with hydrological alteration degrees of 50.86% and 83.27%, respectively. The former parameter increased, but the latter decreased significantly in the post-altered period. To induce the spawning activity of FMCC and CS, appropriate ecological flows and hydrological parameters were proposed. These results showed that during the spawning seasons of FMCC and CS, the hydrological processes of the middle reaches of the Yangtze River changed significantly. Therefore, ecological flow must be ensured through ecological operation of upstream reservoirs to provide suitable spawning conditions in target fish spawning grounds.

## Introduction

River hydrological processes are the primary drivers of river ecosystems, and their natural regimes have been adopted as paradigms for protecting and restoring ecological integrity. River hydrologic variability directly affects aquatic species and abundance^1,2^. Fish are essential aquatic biological resources that are sensitive to changes in flow regime^3,4^, especially during spawning and rearing. They are therefore a good indicator of the health of aquatic ecosystems^5^. In order to protect fish resources, many studies have been conducted to identify the factors that affect fish habitat and lifestyle^6–8^.

The analysis of ecohydrological processes during the fish spawning season can be divided into two categories: mechanism model that considers physical and hydrodynamic conditions^9,10^, and correlation analysis between monitored biomass data and measured ecohydrological indices^11^. The latter has proven to be an excellent method for identifying influencing factors with limited physical and spatial data^12^. For example, several studies have used covariance and regression analysis to infer relationships between hydrologic dynamics and influencing factors such as precipitation, topography, soil, vegetation and climate, etc., in order to characterize hydrologic events in large rivers^13,14^. These studies have shown that the spawning behavior of fish is a response to the number, duration of the water rising process and other ecohydrological and water temperature indices^15^. Peng, et al.^11^ showed that the reduction in peak flows caused by the dams would result in the failure of downstream fish to spawn. Given this challenge, quantitative assessment of changes in characteristic hydrologic parameters^16^ and analysis of ecological flows required by the fish spawning process^2,11,17^ are essential for river habitat restoration.

There are three critical procedures involved in evaluating the hydrologic process change for fish spawning. The first is to determine the hydrologic change year. This can be done in two ways. One is to infer the time to play a role according to the construction schedule of water conservancy projects^18^, and the other is to select a typical period according to the needs of the study to conduct trend analysis on the flow process in the typical period^19^. Recent studies have considered the completion of water conservation projects as an abrupt year^20^, neglecting the long construction period of these projects. In addition, the significantly altered extreme hydrological characteristics of rivers are usually affected by the combination of climatic change and anthropogenic activities^21^. Considering only the construction schedule of water conservancy projects can only describe the macroscopic changing state of rivers^22^. In fact, the water rising process shows the distribution of the flow discharge within a year, rather than the interannual flow. Obviously, trend analysis is more appropriate for evaluating hydrological processes during the fish spawning season. The Mann–Kendall nonparametric test and the sliding t-test are practical tools for analyzing abrupt alteration tests of runoff, temperature, and precipitation^23^. The Mann–Kendall test may produce multiple intersections outside the confidence interval, so the sliding t-test can be used for additional verification. The results of the mutation year selection should then be judged to be reasonable.

The second procedure is the selection of appropriate indicators for the identification of hydrological processes. Changes in hydrological and hydraulic factors associated with river flow, i.e. flow rate, velocity, water quality and hydrological conditions that affect the physiological activity of fish^24^. Ecological flow regimes with specific ecohydrological characteristics (e.g., flow, frequency, duration, timing, and rate of change) have been reported to provide not only oviposition but also the necessary egg drift conditions^25^. On this basis, Qiu et al.^26^ first proposed a group of fish-oriented indicators to describe the characteristics of the rising flow processes, consisting of flow rising and falling edges. These indicators of flow augmentation differ from existing flow regime analyses such as Indicators of Hydrological Alteration (IHA) and Ecologically Relevant Hydrological Indicators (ERHI)^27^, which suggest that only flow pulses of a certain amplitude and duration can effectively stimulate fish spawning^3^. Since previous studies have confirmed that the number, duration, and daily flow increase rate of the water rising process can be used to quantitatively describe the water rising process in a certain period^28^, the date of the first flooding and the initial flow rate are used as additional water rising characteristic parameters in this study.

The final step is to assess the variability of hydrologic indicators. Previous studies have shown that the range of variability approach (RVA) in combination with IHAs can successfully and powerfully reveal flow regime changes^16^. However, they ignored the frequency of the natural flow, which was already altered^29^. Shiau and Wu^30^ proposed a histogram matching method (HMA), which can investigate the changes in hydrological processes between pre-altered and post-altered with a quadratic-form distance. It was selected to evaluate the degree of hydrological alteration in this study.

River flow regimes can be assessed to determine the detrimental effects of water conservation projects and climate change on fish reproduction^31^, which can be further applied to ecological flow design and management^32^. To account for specific river habitat conditions, Yu et al.^33^ revised the variable monthly flow (RVMF) method through scenario analysis using the classification concept of the Tennant method. Książek et al.^34^ proposed an environmental flow assessment method that combines the hydrologic (Tennant) and hydraulic (wetted perimeter) approaches. Wang et al.^9^ developed an improved habitat model that considers both habitat quantity and quality. The model was applied to obtain ecological flow for spawning and rearing of Chinese Sturgeon (CS). Ecological flow assessment using hydraulic rating curves with minimum spawning requirements and their additional criteria, such as the use of indicator species composition and seasonal variation^35^, may result in a more appropriate multicomponent flow regime than fixed minimum flows^36^. In addition, hydraulic-habitat models can be used to determine optimal ecological flows through regression models between zoobenthos or fish species richness and changes in the flow regime^37^. The above studies showed that the altered hydrological process in downstream rivers can be indicated by the degree of proximity of hydrological parameters to their natural state. Similarly, the closer the values of the characteristic parameters are to their natural state, the less the environmental degradation or loss of biodiversity in the affected areas^38^. Therefore, river managers can identify ecological flow for fish habitat restoration in terms of the quantitative relationship between river flow and hydrologic parameters.

Based on the above analysis, it is necessary to identify the changing hydrological processes during the fish spawning period and to recognize the ecological flow associated with characteristic hydrological parameters to provide spawning conditions for the targeted species. However, there is a lack of a systematic framework for evaluating the alteration of hydrologic processes and the required ecological flows during the fish spawning season. This study aims to fill this research gap. The objectives of this study are: (1) to identify the characteristic hydrological parameters of water rising processes by analyzing the daily flow data sets in fish spawning period in each monitoring year; (2) to quantify the hydrological process alteration during fish spawning period by comparing for frequency histograms of each hydrological parameter; (3) to obtain the ecological flow by simulating the natural hydrological processes with the daily ecological flow optimization model. The novelty of this research is to propose a comprehensive method to evaluate the alteration of water rising process and an ecological flow optimization model for fish spawning. This process can analyze hydrological and ecological changes in the dammed river for the protection of river water resources and ecosystem.

## Material and methods

### Study area

**Figure 1:**
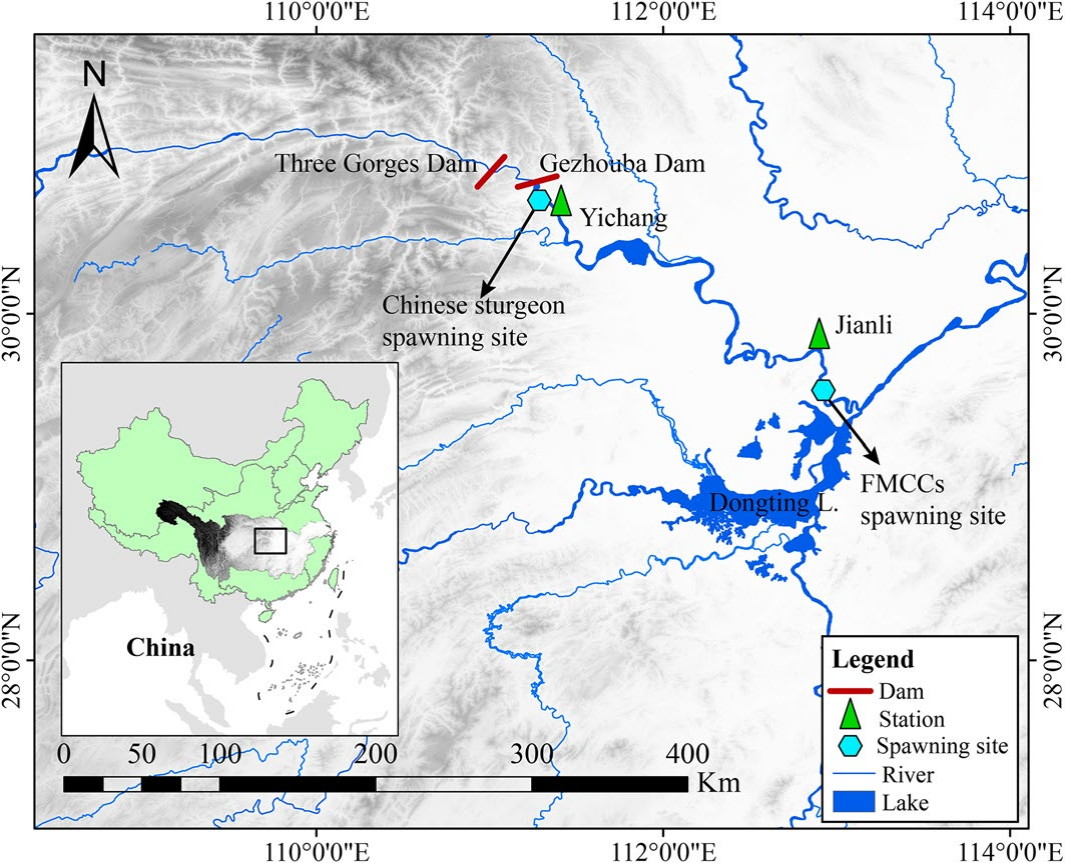
Diagram of the study area. This figure was created using ArcGIS 10.6 software (https://www.esri.com/en-us/arcgis/products) and the review number of China Map is GS (2020) 4634.

Our study focused on a stretch of approximately 420 km from the Three Gorges Dam (TGD) to 70 km downstream of the Jianli hydrologic station in the middle reaches of the Yangtze River (Fig. 1). This covers the critical spawning grounds of CS and Four Major Chinese Carps (FMCC). The Yichang hydrologic station is located 44 km downstream of the TGD and controls river discharge into the middle reaches of the Yangtze River. The Jianli hydrologic station is located approximately 350 km downstream of the TGD.

### Data acquisition

In this study, two types of data were collected. Daily streamflow data for the Yichang (1950–2020) and Jianli (1980–2020) hydrologic stations were obtained from the Changjiang Water Resources Commission (CWRC). Aquatic biological data collected in the field were obtained from the Ecological and Environmental Monitoring Bulletin of the Three Gorges Project (TGP) and published references. Among these data, the annual numbers of CS spawners were observed at the spawning grounds below the Gezhouba Dam during the spawning season^39,40^. The annual runoff of FMCC eggs and larvae was surveyed from April to July in the Jianli section of the middle reaches of the Yangtze River^32^. The homogeneity and reliability of these data have been rigorously tested and controlled by the Hydrological Bureau before being released for analysis.

### Sensitive indicator species

Fish inhabit the headwaters of river ecosystems and thus better reflect the ecological health of rivers. They play an important role in maintaining the integrity of aquatic ecosystems and ensuring the provision of environmental services. The FMCC (black carp (*Mylopharyngodon piceus*), grass carp (*Ctenopharyngodon idellus*), silver carp (*Hypophthalmichthys molitrix*), and bighead carp (*Aristichys nobilis*)) are important economic fish that maintain the genetic diversity of freshwater fish farming in China^7^. The critical period for FMCC spawning is from the end of April to the middle of July^41^. More than 10 FMCC spawning sites have been identified in a 70-km stretch of the river downstream of Jianli, and the spawning size accounts for 42.7% of that of the entire Yangtze River^42^.

The CS (*Acipenser sinensis Gray*) was listed as a critically endangered species by the International Union for Conservation of Nature in 2014. It is of great importance for the development and rationalization of wildlife resources and the maintenance of ecological balance to protect and save this rare and endangered living fossil. The interception of the GD cut off the spawning migration route of the CS. New spawning sites appeared 5 km downstream of the GD. From October to November, CS spawn and hatch annually in the Yichang Section^43^. During this period, runoff and water temperature fluctuations affect gonad development and result in spawning delays^4,32^.

### Analysis of water rising process alteration

#### Detection of mutation year

The hydrological mutation years are the points in time when significant changes in hydrological parameters occur. They divide the study period into two sub-periods: pre-altered (before the mutation year) and post-altered (after the mutation year). As the runoff in the middle and lower Yangtze River is affected by overlapping multiple water conservancy projects and climate change, several abrupt change years may occur. Therefore, two techniques (Mann–Kendall test and sliding t-test) were selected for the clarification and verification of the trends of the hydrological process regime.

According to the Mann–Kendall test, a mutation year in the flow series can be observed when the progressive series ${UF}_{k}$ and the retrograde series ${UB}_{k}$ intersect in a certain year at the significance level of $\mathrm{\alpha }=0.05$. The detailed calculation procedures are described in the reference^44^.

The sliding t-test divides the time series into two sub-series and tests for mutations by comparing the mean values of the sub-series. In order to avoid the shift of the mutation point caused by the length of the series, the sub-series are tested in different time steps. For detailed computational procedures, see the literature^45^.

#### Water rising process denoising

**Figure 2:**
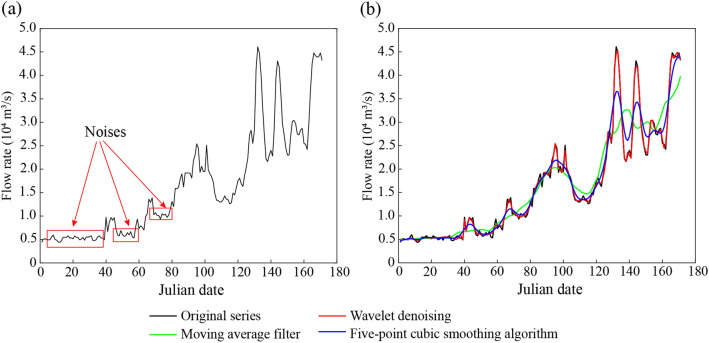
An example of the daily flow series with and without denoising processing (**a**) original series; (**b**) comparison of the original series and the denoised series.

The water rising process in this study refers to a complete wave with a certain amplitude and duration, which can effectively stimulate fish spawning^3^. Daily flow series exhibit high fluctuations in addition to nonlinear and seasonal signals (Fig. 2a), and the noise of the time series cannot be perceived by these native fish, affecting the accuracy of identifying characteristic parameters^28^. Therefore, the denoising of the original time series to eliminate the noise is a prerequisite for the identification of the water rising process and the extraction of the characteristic parameters. The raw data showed unexpected deviations, especially the maximum and minimum flow values, when wavelet denoising^46^, moving average filter^47^ and five-point cubic smoothing processing^48^ were used to smooth the raw data (Fig. 2). Therefore, we proposed an improved water rising process denoising method based on constraint conditions. The detailed algorithm is as follows:

(1) Water rising process identification and endpoint selection.

The first derivative is used to identify all the extreme points of the original data. Since the beginning and end of the sequence are monotonically increasing or decreasing, the possible water rising process at these two points cannot be identified using the first derivative. We then added ${a}_{0}$ and ${a}_{n+1}$ at the beginning and end of the original sequence to obtain the paired maxima and minima points [S, L] (Eq. (1)). The flow between the minima S and the maxima L is a water rising process.

1 $$A_{i} = a_{i} - a_{i - 1} , i = 1,2, \ldots ,n,\,\,n + 1;\,\,\,\,a_{0} = a_{2} , a_{n + 1} = a_{n - 1}$$

If ${A}_{i}>0$ and ${A}_{i+1}<0$, then ${a}_{i}$ is the maxima; If ${A}_{i}<0$ and ${A}_{i+1}>0$, then ${a}_{i}$ is the minima.

(2) Amplitude and duration of the water rising process.

**Figure 3:**
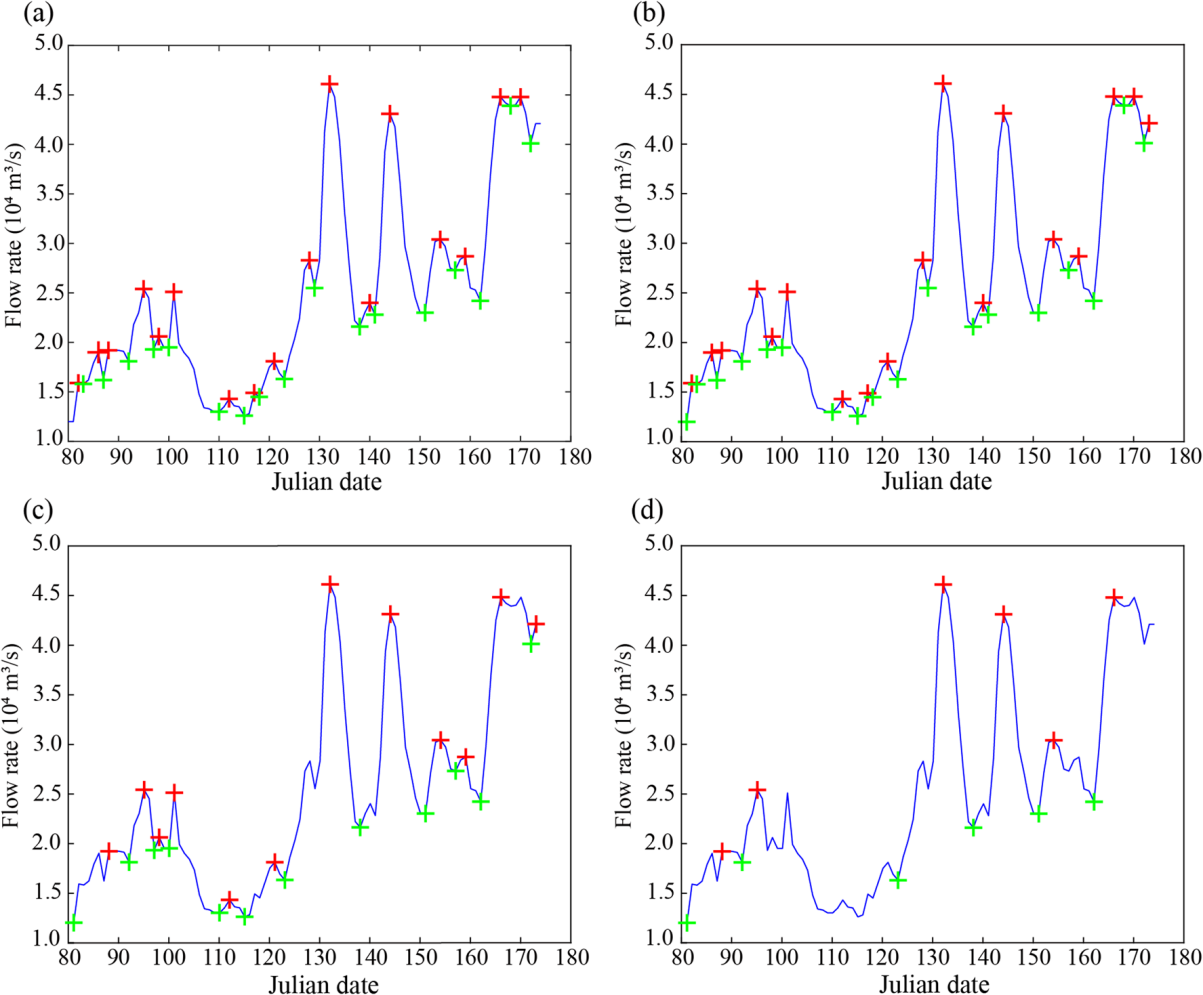
Procedures for the water rising process identification (**a**) initial water rising process; (**b**) after adding endpoints; (**c**) after processing the magnitude and duration of the water rising process; (**d**) after processing the magnitude and duration of the water falling process.

Increasing flow with a specific amplitude and duration can effectively stimulate fish gonad development^4^. The amplitude of the flow increase refers to the subtraction of the minimum from the maximum of an extreme pair. If the rising amplitude is less than a certain percentage of the maximum rising amplitude for the year (set to 20% in this study)^28^, the water rising process is considered to be a minor invalid fluctuation and will be abandoned (Fig. 3).

(3) Amplitude and duration of the water falling process.

For a water rising process to be perceived by the fish, there must be a time lapse between two adjacent high water peaks^49^. Accordingly, the paired extremes [${S}_{i},{L}_{i}$] identified in step (1) can be refined according to the amplitude and duration of the water falling process (in this study, the minimum falling amplitude is set to 10% of the initial rising amplitude, and the minimum falling duration is two days)^50^. The maximum value among ${L}_{i}$, ${L}_{i-1}$, and ${L}_{i+1}$ is retained as the flood peak point if three adjacent paired extremes [${S}_{i-1},{L}_{i-1}$], [${S}_{i},{L}_{i}$], and [${S}_{i+1},{L}_{i+1}$] do not satisfy the amplitude and duration constraints of the water falling process.

#### Definition of characteristic hydrological parameters

(1) Water rising processes count $N$

In a certain period, the complete wave between the minimum flow and its adjacent maximum flow is called a water rising process, which has a certain magnitude and duration^28^. The number of water rising processes in the spawning season is defined as the water rising processes count $N$.

(2) Water rising process duration $\overline{T }$

2 $$\overline{T} = \left( {\mathop \sum \limits_{i = 1}^{N} T_{i} } \right)/N$$

where $\overline{T }$ represents the average duration of water rising processes $\left(d\right)$, and ${T}_{i}$ is the duration of the ${i}^{th}$ water rising process $(d)$.

(3) Daily flow increase rate $\overline{\eta }$

3 $$\overline{\eta } = \left( {\mathop \sum \limits_{i = 1}^{N} \frac{{\max \left( {Q_{i} } \right) - \min \left( {Q_{i} } \right)}}{{Day\left| {\max \left( {Q_{i} } \right)} \right. - Day\left| {\min \left( {Q_{i} } \right)} \right. + 1}}} \right)/N$$

where $\overline{\eta }$ is the average daily flow increase rate $\left({m}^{3}/(s\cdot d)\right)$, ${Q}_{i}$ represents the flow rate of the corresponding section $\left({m}^{3}/s\right)$, and ${\text{Day|}}\max Q_{i}$ and ${\text{Day|}}\min Q_{i}$ are the Day of the Year Calendar dates of the occurrence of maximum and minimum flow in a water rising process, respectively.

(4) Date of the water rising process $D$

When the TGR was ecologically scheduled by generating an excellent water rising process in late June each year, a peak runoff of FMCC eggs and larvae would occur in the Jianli Section^51^. This suggests that changes in the timing of rising flows will also disrupt hydrological signals for fish migration and spawning^39^. With this, we defined the Day of the Year Calendar date when the first water rising process in the spawning season starts as the date of the first water rising process $D$ in this study, which would affect the occurrence of the mass spawning quantity.

(5) Initial water rising flow $F$

Initial water rising flow is the flow at the beginning of the first water rising process; it is also a critical indicator of fish production by inducing the timing of spawning^28^.

#### Assessment of hydrological process alteration

The distance formula in HMA can quantify the total (dis)similarity of two histograms by comprehensively considering the inter-class correspondence and cross-class information^52^.

First, the number of histogram classes for each parameter can be determined as follows:

4 $${n}_{c}=\frac{r\cdot {n}^{1/3}}{2\cdot {r}_{d}}$$

where ${n}_{c}$ is the number of classes, $r$ is the difference between the maximum and minimum values, $n$ is the total number of each hydrological parameter, and ${r}_{d}$ is the difference between the first and third quartile values.

The quadratic-form distance for the ${m}^{th}$ parameter is defined as follows:

5 $${dQ}_{m}\left(H,K\right)=\sqrt{{(\left|H-K\right|)}^{T}\cdot A\cdot (\left|H-K\right|)}\cdot 100\mathrm{\%}$$

where $H={({h}_{1},{h}_{2}, \dots ,{h}_{{n}_{c}})}^{T}$ and $K={({k}_{1},{k}_{2}, \dots ,{k}_{{n}_{c}})}^{T}$ are frequency vectors of the histogram of each hydrological parameter in pre-altered and post-altered, respectively; and $\left|H-K\right|$ represents the absolute value vector of ($H-K$). $A=[{a}_{jk}]$, where ${a}_{jk}$ denotes the similarity between classes $j$ and $k$:

6 $${a}_{jk}={(1-\frac{{d}_{jk}}{{d}_{max}})}^{\alpha }$$

where ${d}_{jk}= |{v}_{j}-{v}_{k}|$ is the absolute distance between classes $j$ and $k$, ${v}_{j}$ and ${v}_{k}$ are the mean values of classification interval for classes $j$ and $k$, respectively; ${d}_{max}$ is the maximum value of ${d}_{jk}$, and $\alpha$ is a constant between 1 and ∞ (a linear function with $\alpha$=1 was adopted in this study)^29^. Note that when ${dQ}_{m}\left(H,K\right)$ exceeds 50%, it represents a significant change of the ${m}^{th}$ parameter in the water rising process.

### Ecological flow optimization model

(1) Objective function.

For each hydrological year, the objective functions can be constructed by determining the proximity of the five characteristic parameters ($N$, $\overline{T }$, $\overline{\eta }$, $D$ and $F$) to their natural state values by optimizing the river flow during the fish spawning period. The formulas are as follows:

7 $$\mathrm{min}f=\sum_{i=1}^{m}{w}_{i}\cdot \sqrt{{\left(\frac{{P}_{i}-{P}_{0, mean}^{i}}{{P}_{0, max}^{i}}\right)}^{2}}$$

where ${w}_{i}$ is the weighting factor of each characteristic parameter, which can be obtained by the proportion of the quadratic-form distance ${w}_{i}=\frac{d{Q}_{m}}{\sum_{i=1}^{m}(d{Q}_{m})}$ for each characteristic parameter during CS and FMCC spawning period, respectively. $m$ is the total number of characteristic parameters. ${P}_{i}$ is the corresponding characteristic hydrological parameter value, and ${P}_{0, mean}^{i}$ and ${P}_{0, max}^{i}$ are the annual mean and maximum values of each characteristic parameter in wet, normal, and dry years in the pre-altered, respectively.

The uninfluenced characteristic parameters indicate the suitable water rising process for FMCC and CS spawning in different hydrological years. According to the Pearson III fitting curve of the annual mean streamflow, the years of the pre-altered period with frequencies ϕ ϵ [37.5%, 62.5%] were considered as normal years, ϕ < 37.5% as wet years, and ϕ > 62.5% as dry years.

(2) Constraint

8 $${Q}_{i}^{sub-min}\le {Q}_{i}\le {Q}_{i}^{sub-max}$$

The sub-maximum discharge ${Q}_{i}^{sub-max}$ and the sub-minimum discharge ${Q}_{i}^{sub-min}$ in post-altered were used as input limits for each hydrological year to maintain the primary function of the existing water conservancy projects and the ecological safety of the downstream river.

(3) NSGA-III-EO algorithm.

Based on the original NSGA-II algorithm, Deb and Jain^53^ incorporated the decomposition and selection mechanism and proposed NSGA-III. Bi and Wang^54^ introduced elimination operators into the mutation process and offered an improved non-dominated sorting genetic algorithm III (NSGA-III-EO), which can provide effective, reliable, and controllable solutions for constrained multi-objective optimization problems^55^. We used this method to solve the constructed ecological flow optimization model. Its general framework is presented in Supplementary Information, Table A1.

**Figure 4:**
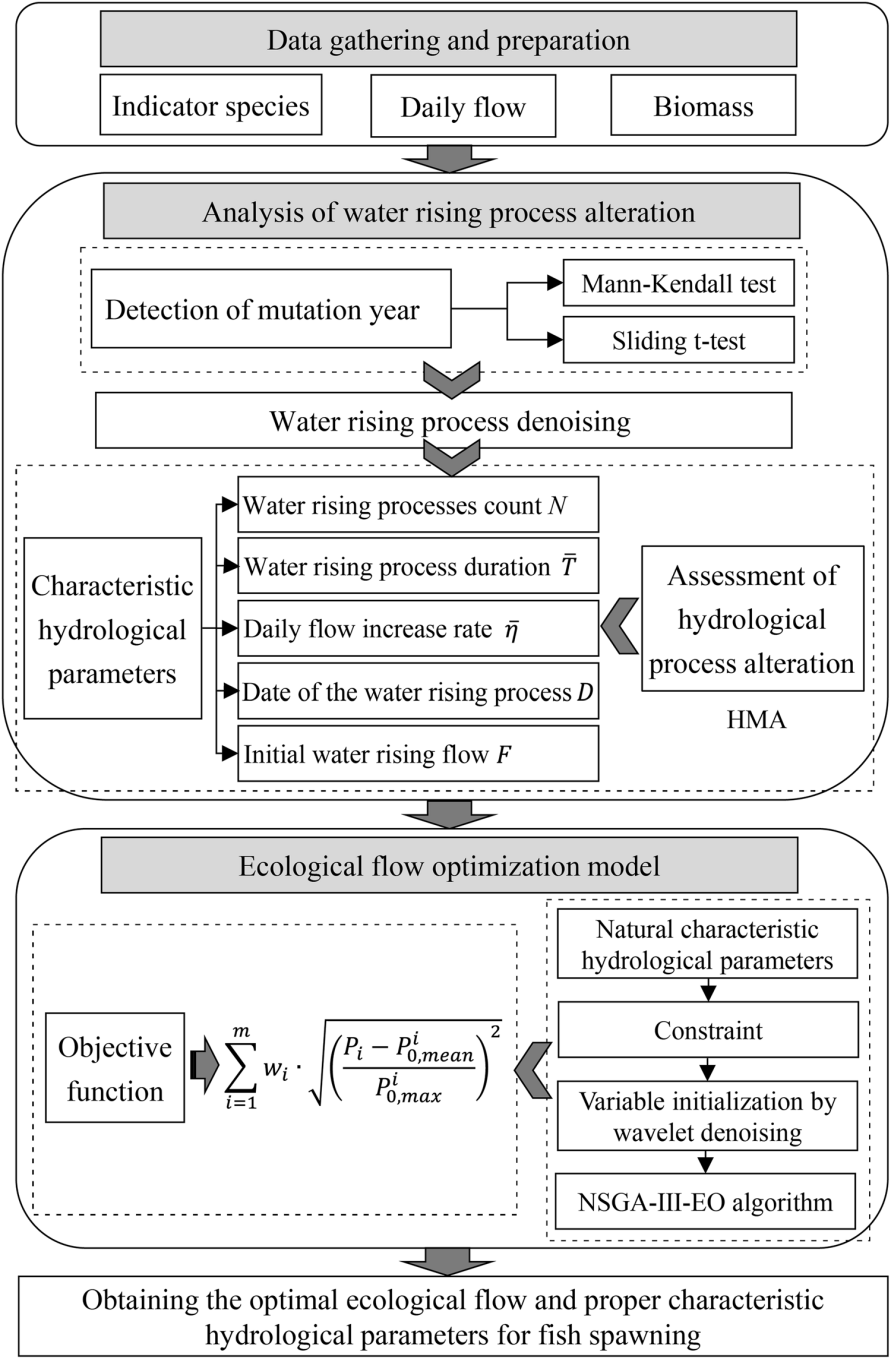
The technical workflow of this study.

Daily flows during the FMCC and CS spawning periods for Yichang and Jianli were the decision variables of the ecological flow optimization model. The model was solved using the NSGA-III-EO algorithm with a population size of 800 and 60 iterations. As the daily ecological flow sequences generated in the variable initialization in NSGA-III-EO showed frequent and pronounced fluctuations, they did not correspond to the actual river flow regime. Therefore, wavelet denoising was performed for each chromosome obtained by variable initialization in NSGA-III-EO to prevent high-frequency fluctuations^46^. Then, the optimized daily ecological flow for maintaining fish spawning was obtained for different hydrological years in Yichang and Jianli sections, respectively. The overall technical workflow is shown in Fig. 4.

## Results

### Abrupt change year identification

**Figure 5:**
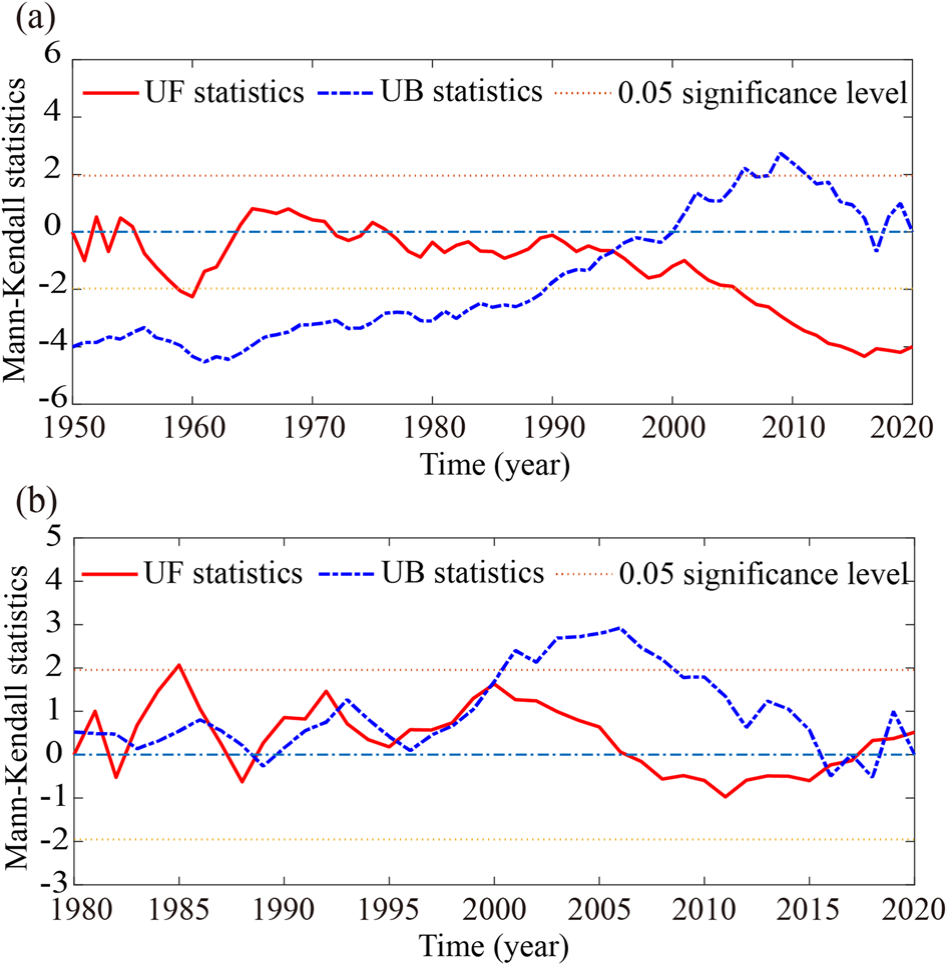
Mann–Kendall tests for mean daily streamflow during the fish spawning season at two stations. (**a**) Yichang (10.1–11.30); (**b**) Jianli (4.21–7.20).

In the Mann–Kendall test, the intersection of the UF curve and the UB curve within the interval of the significance level are the abrupt years^1^. Figure 5 shows the variation of the Mann–Kendall values of the mean daily flow during the fish spawning season in each monitoring year in Yichang and Jianli. From Fig. 5a, the UF and UB curves of the mean daily flow during the CS spawning season in Yichang intersected at one point (1995) at the confidence level of $\mathrm{\alpha }=0.05$, which can be considered as the abrupt change year of the hydrological process of the CS spawning period in the Yichang section. In Fig. 5b, for a given significance level of $\mathrm{\alpha }=0.05$, the UF and UB curves have eight intersection points within the confidence intervals. These intersections can be considered as abrupt years when hydrological processes changed during FMCC spawning season in Jianli section.

The mutation years detected by Mann–Kendall test and sliding t-test methods are shown in Table 1. Based on the Mann–Kendall test and sliding t-test, the mutation year of the mean daily flow in the FMCC spawning season in Jianli occurred in 2000, but the mutation year detected by the two methods in Yichang is not a uniform year. We then investigated the adaptability of the two methods for detecting change points in hydroclimate time series. As mentioned by Xie et al.^56^, the large coefficient of variation (Cv) in a time series would cause the difficulty in detecting the abrupt change point. The Cv value of the mean daily flow of the CS spawning season in Yichang and the FMCC spawning season in Jianli are 0.20 and 0.10, respectively. In Xie’s experiments^56^, when the variation coefficient Cv of the time series is 0.2, the sliding t-test maintains its superior performance and has an efficiency of more than 90%, but the efficiency of the Mann–Kendall test quickly decreases by less than 30%. They concluded that sliding t-test had better performance than the Mann–Kendall test and was recommended for change point detection. In addition, other researchers^57^ have found that the Mann–Kendall and Pettitt methods are not suitable for testing multiple mutation points, while the sliding t-test and Yamamoto methods are more accurate for testing multiple mutation points. According to the above analysis, the abrupt years of the mean daily flow series of Yichang in the CS spawning season would be determined by the sliding t-test method. In summary, in this study, the year 2000 is selected as the abrupt change year of the daily flow changes in the fish spawning season in Yichang and Jianli. Therefore, the period between the initial monitoring year and 1999 was defined as the pre-altered period, and the period between 2000 and 2020 was considered as the post-altered period.

**Table 1:** Mutation years of the mean daily flow during the fish spawning season at the two hydrologic stations.

Hydrologic stations	Mann–Kendall test (significant level = 0.05)	Sliding t-test (significant level = 0.01)
CS spawning season at Yichang (10.1–11.30)	1995	1960, 1998, 2000, 2001, 2005
FMCC spawning season at Jianli (4.21–7.20)	1980, 1981, 1986,1995, 2000, 2016, 2017, 2019	1985, 1988, 2000, 2002, 2011

**Figure 6:**
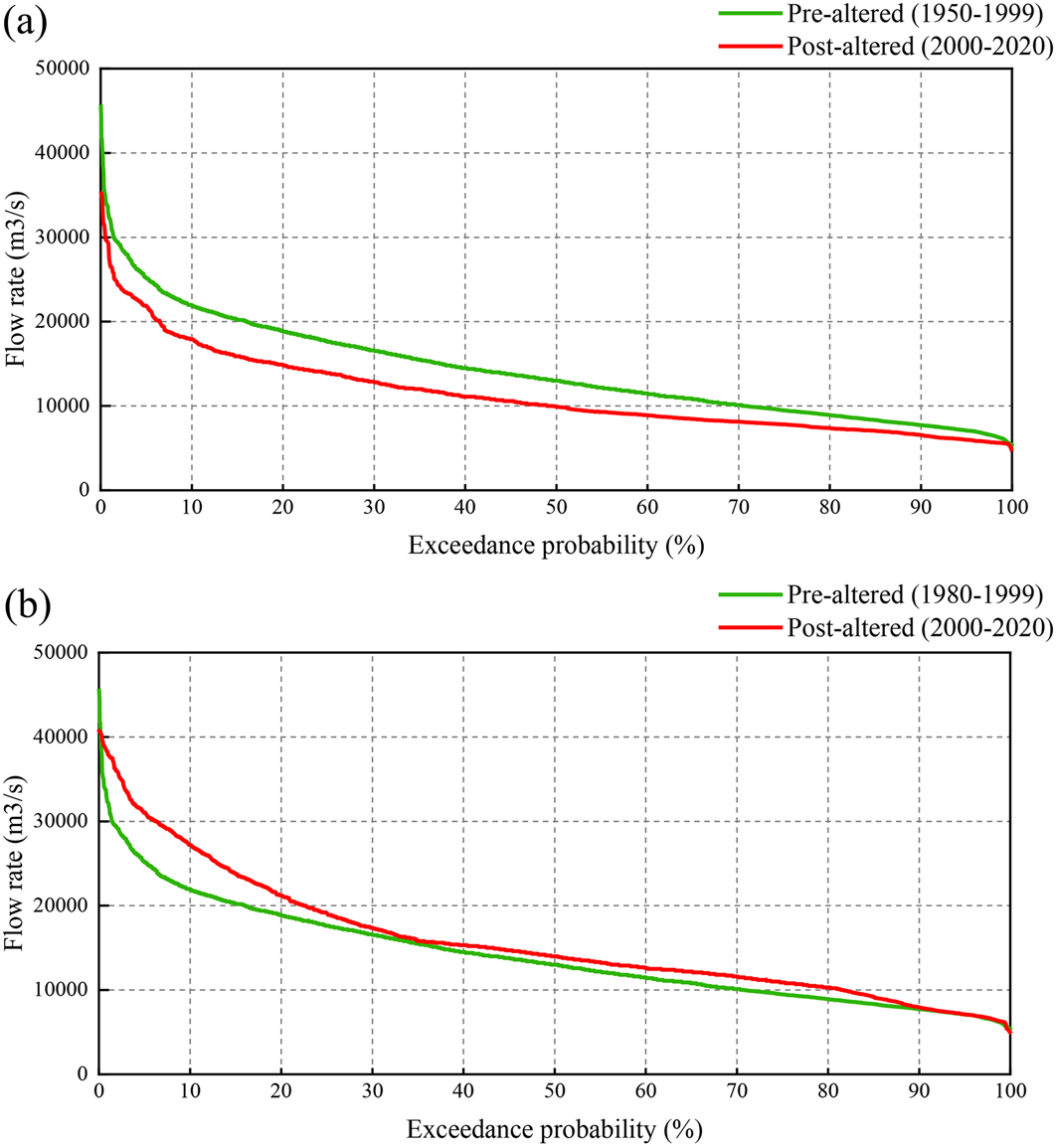
Flow duration curves of mean daily flow in the fish spawning season at two stations over different periods. (**a**) Yichang; (**b**) Jianli.

A flow duration curve (FDC) presents the daily flow values and their corresponding exceedance probabilities in a simple graphical representation of the flow variability^58^. An FDC was then plotted using daily flow records during the fish spawning season in the pre-altered and post-altered periods to further confirm the mutation year. Figure 6 shows the daily FDCs for the CS spawning period at Yichang and the FMCC spawning period at Jianli in the pre- and post-altered periods. The magnitude of daily flow in the post-altered period was generally smaller than that in the pre-altered period at Yichang in the CS spawning season (Fig. 6a). In addition, the magnitude of daily flow during the FMCC spawning season in the post-altered period at Jianli was almost higher than that in the pre-altered period. Specifically, the difference between high flow values for less than 30% exceedance probability is high, and that of after the 30% percentile of low daily flow values is not significant (Fig. 6b).

### Water rising process alterations during fish spawning seasons

**Figure 7:**
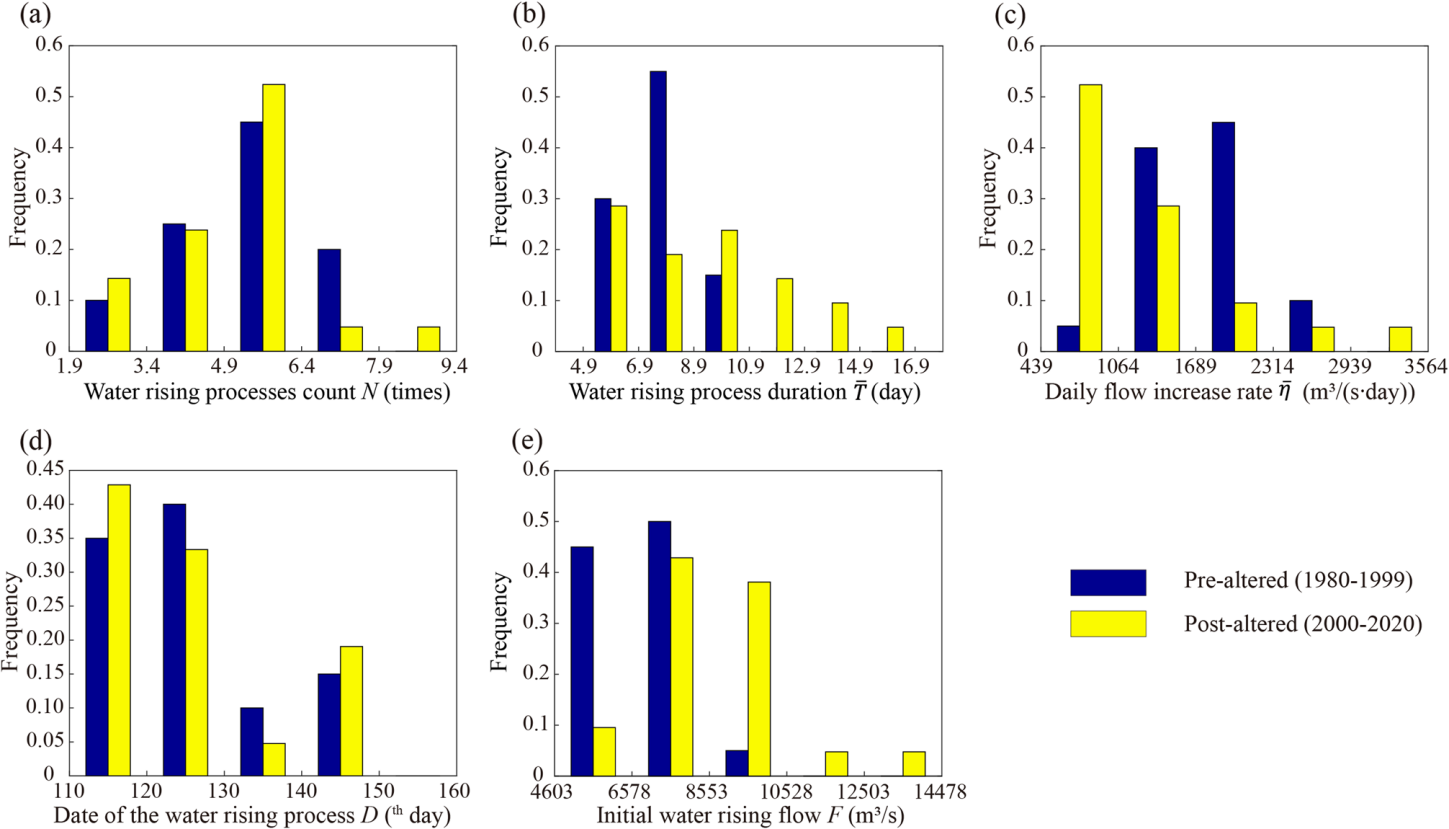
Pre- and post-altered period frequency histograms of characteristic hydrological parameters during FMCC spawning period for Jianli station. (**a**) water rising processes count; (**b**) water rising process duration; (**c**) daily flow increase rate; (**d**) date of water rising process; (**e**) initial water rising flow.

**Figure 8:**
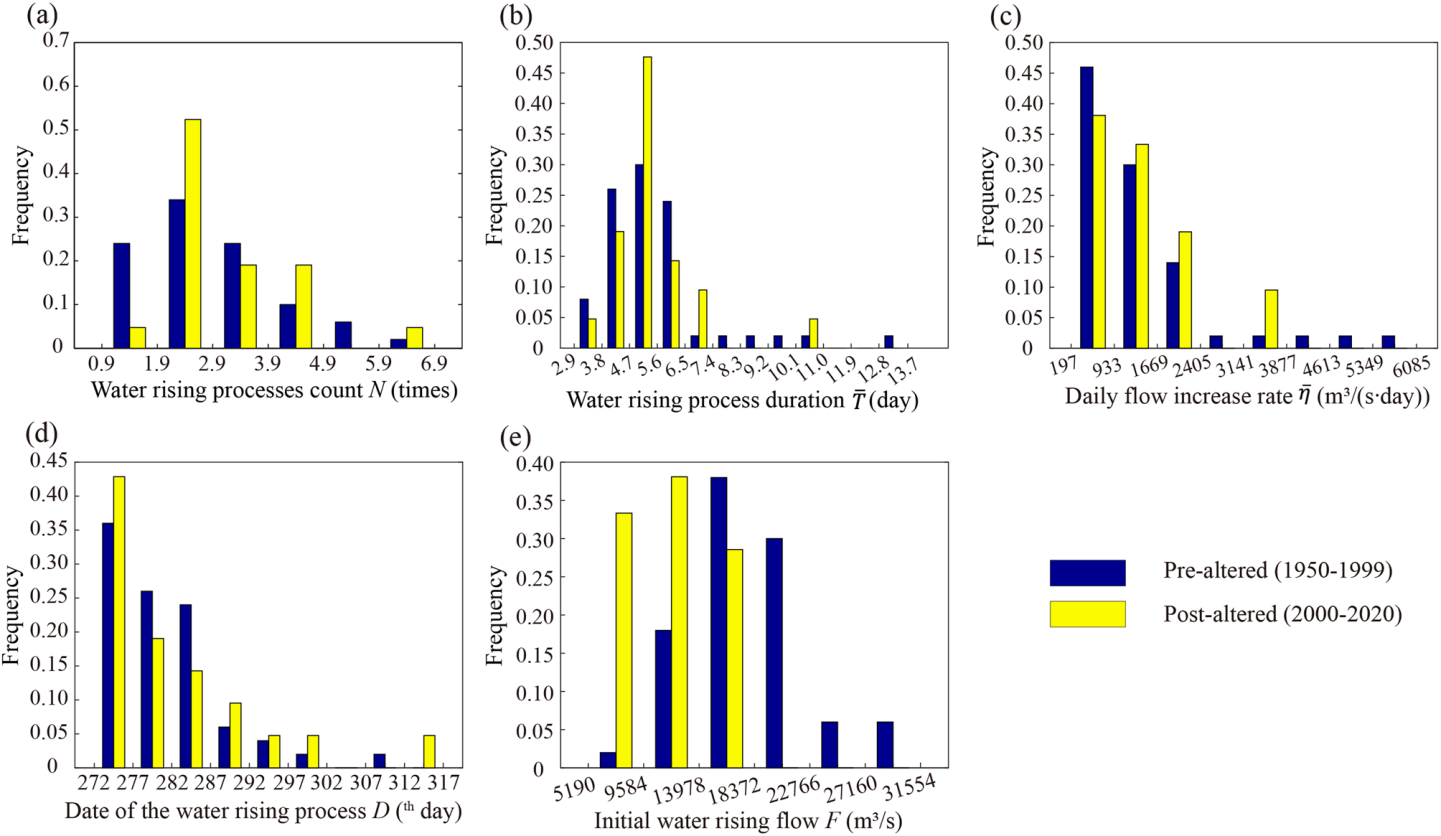
Pre- and post-altered period frequency histograms of characteristic hydrological parameters during CS spawning period for Yichang station. (**a**) water rising processes count; (**b**) water rising process duration; (**c**) daily flow increase rate; (**d**) date of the water rising process; (**e**) initial water rising flow.

Table 2 shows the hydrological process variations during the spawning season of FMCC and CS at their spawning grounds in the pre- and post-altered periods. The frequency histograms of characteristic parameters for Jianli and Yichang stations are shown in Figs. 7 and 8.

**Table 2:** Characteristic parameter values and HMA distance for Jianli and Yichang stations.

Characteristic parameters	During the FMCC spawning season at Jianli (4.21–7.20)			During the CS spawning season at Yichang (10.1–11.30)		
	Mean in pre-altered: (20 years)	Mean in post-altered: (21 years)	$d{Q}_{m}$: quadratic-form distance (%)	Mean in pre-altered: (50 years)	Mean in post-altered: (21 years)	$d{Q}_{m}$: quadratic-form distance (%)
$N$(times)	5.10	4.90	27.35	2.46	2.71	50.86
$\overline{T }$(day)	7.80	9.38	63.10	5.57	5.57	49.06
$\overline{\eta }$ $\left({\mathrm{m}}^{3}/(\mathrm{s}\cdot \mathrm{d})\right)$	1730.18	1264.26	86.50	1361.51	1328.06	25.61
$D$(^th^ day)	126	124.81	12.10	281.34	283.19	30.02
$F$(${\mathrm{m}}^{3}/\mathrm{s}$)	6694	8510	70.16	17,680	11,429.05	83.27

The water rising process duration, daily flow increase rate, and initial water rising flow changed significantly at Jianli, with hydrological alteration of 63.10%, 86.50%, and 70.16%, respectively (Table 2). The water rising process duration was clustered in a low-value interval (Fig. 7b), with a frequency of 0.85 in the pre-altered range of [4.9, 8.9]. While the frequency in this range was 0.48 in post-altered, and the frequency of water rising process duration in post-altered ranged in [8.9, 16.9] was more than 0.5, indicating a significant increase in the FMCC spawning season. For the daily flow increase rate (Fig. 7c), the frequency in [439, 1064] in pre-altered was 0.05, and the frequency in this range was 0.52 in post-altered, indicating that its value decreased significantly in post-altered. In addition, the initial water rising flow during the spawning period of FMCC in Jianli increased significantly (Fig. 7e). The frequency between 6578 and 10,528 in the pre-altered was 0.55, but increased to 0.81 in the post-altered. The increased initial water rising flow may cause a delay in appropriate rising water signals for fish migration^39^. The decreased daily flow increase rate and the increased initial water rising flow at Jianli might be the main hydrological factors unfavorable for FMCC spawning in the post-altered period^59^.

Similarly, the changes in hydrological parameters were assessed at the Yichang section during the CS spawning period. The most obvious altered parameters were the water rising processes count and the initial water rising flow, with hydrological alteration of 50.86% and 83.27%, respectively. Figure 8 shows the frequency distributions of the five characteristic parameters in Yichang during the CS spawning season in the pre-altered and post-altered periods. In the pre-altered period, the cumulative frequencies of the water rising processes count ranging from [0.9, 1.9] and [1.9, 4.9] were 0.24 and 0.68, respectively. In post-altered, the corresponding frequencies were 0.05 and 0.90, respectively, representing a significant increase in the water rising count during the CS spawning season (Fig. 8a). For the initial water rising flow, the cumulative flow frequency between 5190 and 13,978 m^3^/s was 0.20 in pre-altered. However, in the post-altered, the frequency was 0.71 (Fig. 8e), representing a sharp decrease in the initial water rising flow during the spawning period of CS. The analysis results showed that three characteristic hydrological parameters were highly altered during the spawning period of FMCC, and two characteristic parameters were highly altered during the spawning period of CS. This indicates that the process of water rising during the fish spawning season has changed and there is an urgent need to restore fish spawning conditions by adjusting the ecological flow regime.

### Ecological flow for fish spawning

**Figure 9:**
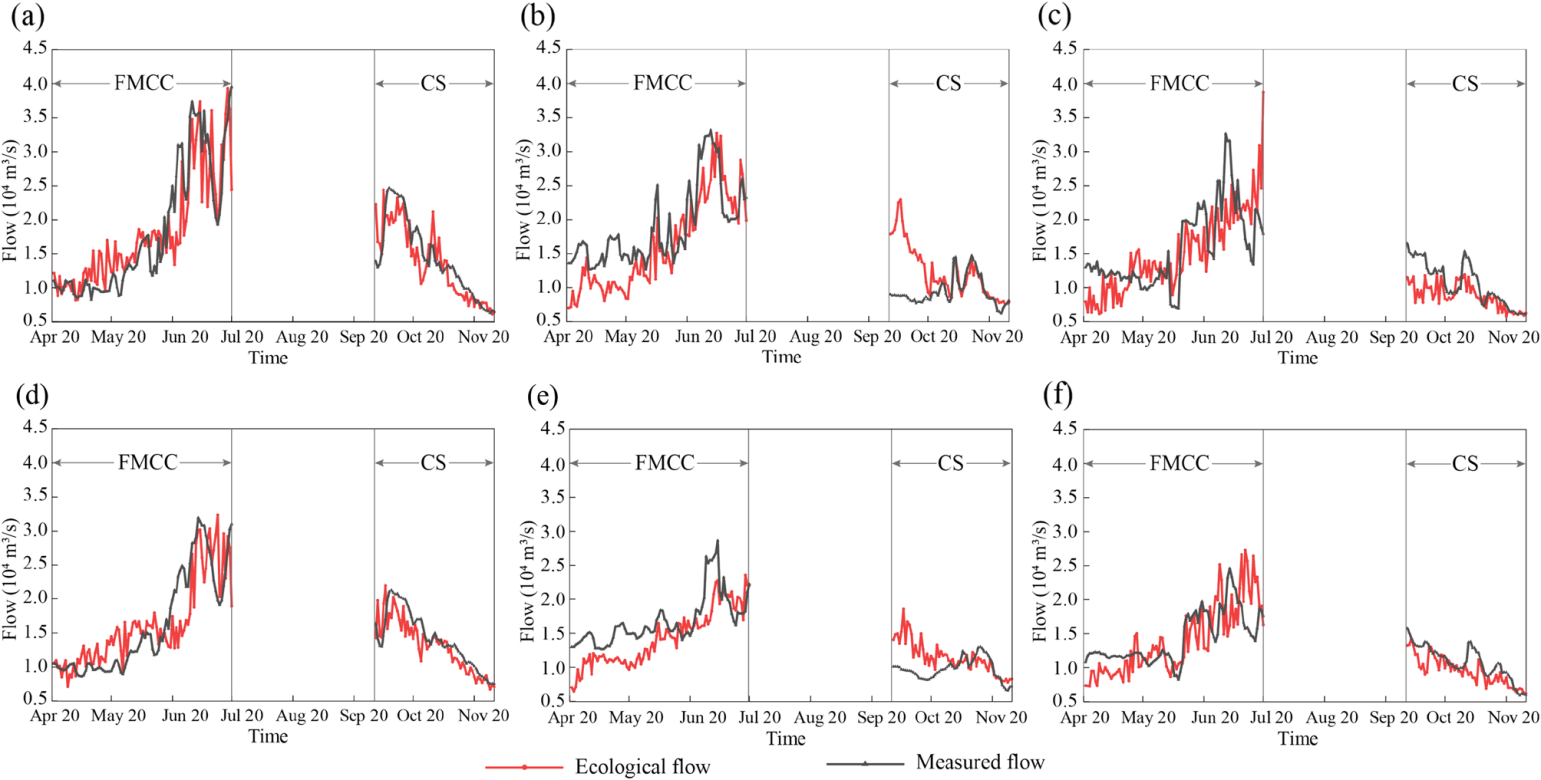
Ecological flow and measured flow for Yichang and Jianli during the CS and FMCC spawning period in the three typical years (**a**–**c** are Yichang in wet, normal, and dry years, respectively; **d**–**f** are Jianli in wet, normal, and dry years, respectively).

The daily ecological flow during the fish spawning period was obtained by simulating the pre-altered hydrological characteristics parameters. The Pearson III curve fitting method was used to divide the post-altered period (2000–2020) into wet, normal, and dry years (Supplementary Information, Table A2). From the three groups of typical hydrological years, the years closest to the present were selected. Accordingly, the years 2020, 2016, and 2015 were chosen as wet, normal, and dry years to demonstrate the optimized ecological flow and the measured flow (Fig. 9).

It can be seen from Fig. 9 that the increases and decreases in daily flow at Yichang and Jianli were generally synchronous within the same hydrological year, indicating the consistent changing trends of the intra-year flow regime. During the reproduction period of FMCC in normal and dry years, the comparison of the optimized ecological flow with the measured flow showed that a flood peak of a certain magnitude is required to slightly decrease at the breeding site during the early spawning stage and slightly increase later^60^. During the spawning season of the CS, the actual flow and the ecological flow showed significant changes, especially in the normal years (Fig. 9b,e). The spawning period of CS coincides with the storage period of the reservoir. Therefore, the TGR should make full use of the small and medium floods at the end of the flood season and start impoundment as early as possible under the premise of ensuring flood control safety, so that the impoundment process in October is as smooth as possible. Then, during the breeding season of CS, the flow at the study site in wet and dry years should decrease slightly compared to the natural release, and the flow in October in a normal year should increase to a higher level than measured. The optimized daily ecological flow can guide upstream reservoirs to perform ecological operations and provide suitable spawning conditions in target fish spawning areas.

## Discussion

### Effects of water rising process on spawning activity

**Figure 10:**
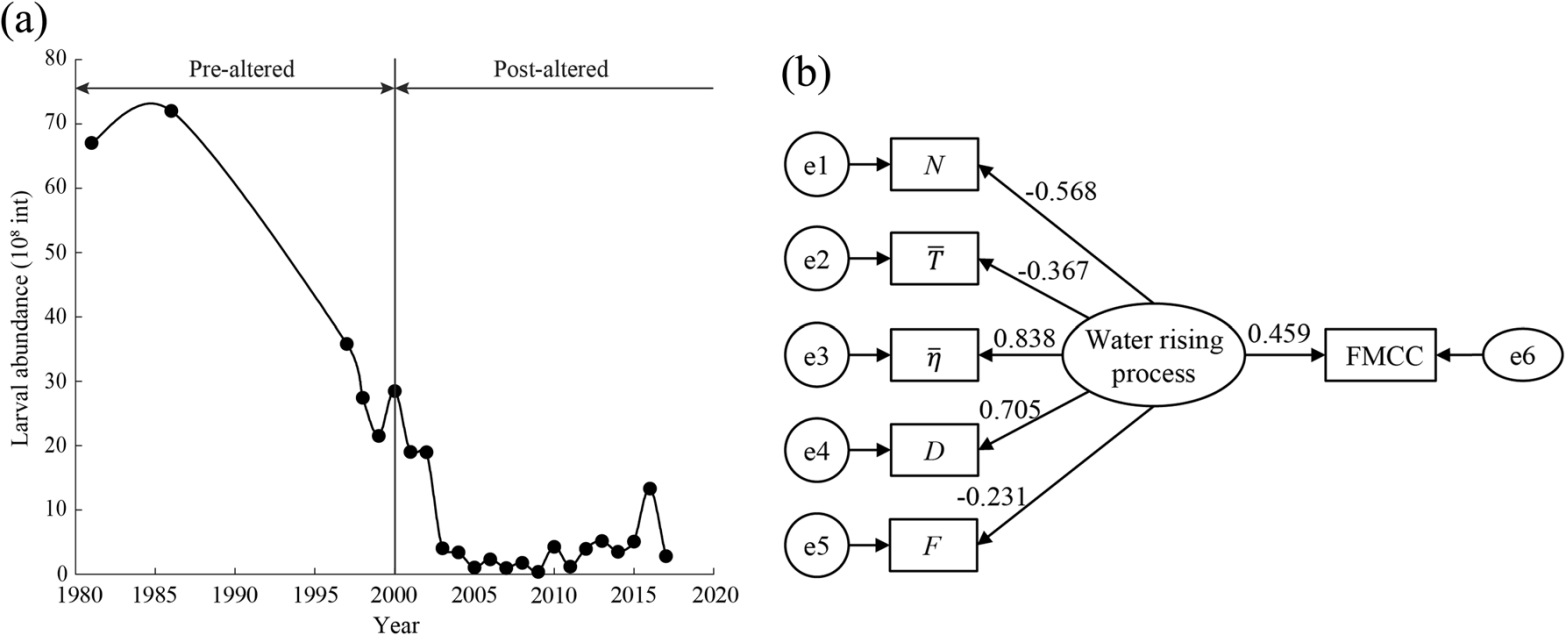
Effects of water rising process on FMCC. (**a**) The breeding scale of FMCC at Jianli; (**b**) standardized path diagram of the structural equation model.

Channel hydrological changes affect the ecological conditions in downstream reaches, including the size, distribution, reproduction, and migration of aquatic organisms^61^. Figure 10a shows the population size of FMCC in Jianli before and after the hydrological abrupt change year. It can be seen that the breeding size of FMCC decreased significantly after 2000. Since 2010, the Yangtze River Fisheries Research Institute and the local government have been continuously releasing FMCC parent fish in this section of the river^62^. And the TGP has been simulated to maintain the duration, magnitude and daily increase rate of the natural flow through ecological operation since 2011. The above measures have promoted the recovery of the FMCC population.

Next, the structural equation model (SEM) was used to investigate the direct and indirect effects of five hydrological parameters on the egg seedlings of FMCC (Fig. 10b). In SEM, the normalized regression weighted values between the potential variables indicate the direct effect value of the latent variables, and the normalized regression weighted value between the indicator variable and the latent variable represents the factor load^63^. The probability level is 0.531, and each goodness of fit indicator meets its criteria (Supplementary Information, Table A3), rejecting the hypothesis that the data deviate from the model; thus, the model works well.

Figure 10b showed that the water rising process alteration positively affected the FMCC egg seedling runoff with a direct effect value of 0.459. The factor loads of $N$, $\overline{T }$, $\overline{\eta }$, $D$, and $F$ were -0.568, -0.367, 0.838, 0.705, and -0.231, respectively. The absolute value of the factor loads of $N$, $\overline{\eta }$, and $D$ were more significant than 0.5. This indicates that they are more important in promoting the reproduction of FMCC than the other two parameters^64^. Combined with the altered characteristic parameters identified in the evaluation of hydrological process changes, the main negative impact of the water rising process on FMCC spawning was the sharp decrease in $\overline{\eta }$ at Jianli. Recent studies have also shown that peak discharge, cumulative increasing discharge in fish perception, and average daily increasing discharge are the most important eco-hydrological indicators of FMCC spawning^65^.

**Figure 11:**
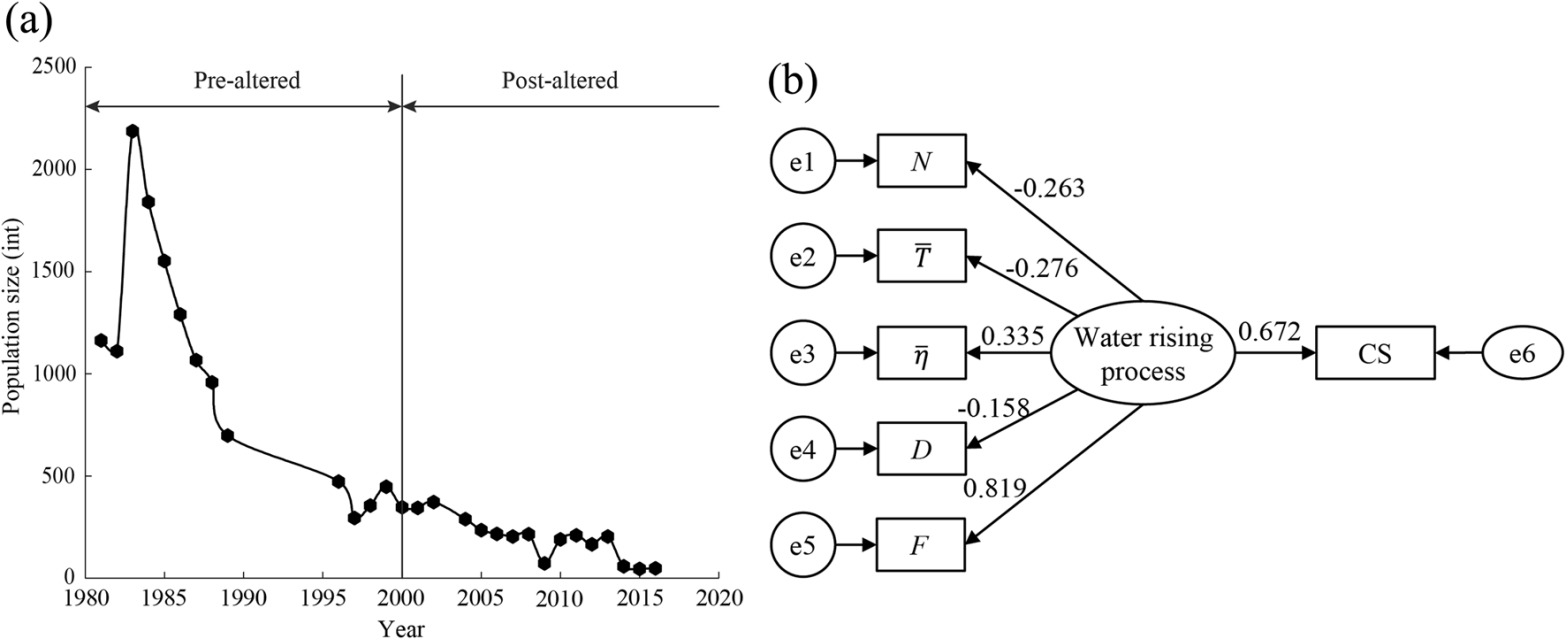
Effects of water rising process on CS. (**a**) The breeding population size of CS in Yichang; (**b**) standardized path diagram of the structural equation model.

Figure 11a shows the reproductive population size of CS in the Yichang section in the pre- and post-altered periods. In the Yangtze River, the construction of the GD has blocked the upstream migration route of the CS since 1981, resulting in an ‘‘aggregation effect’’ that has led to increased density in the Yichang segment^66^. The population began to decline from over 1,000 in 1984–1987 to 347 in 2000. After 2000, the breeding population size of CS on the spawning grounds continued to decline significantly, further confirming our identified mutation year.

Figure 11b shows the direct and indirect effects of the water rising process on the breeding population size of CS. In SEM, the probability level is 0.362; each goodness of fit indicator meets its criteria (Table A4), indicating that the model works well. The direct effect value of the water rising process on the change in CS breeding population size was 0.672, and the factor loads of $N$, $\overline{T }$, $\overline{\eta }$, $D$, and $F$ on the water rising process were − 0.263, − 0.276, 0.335, − 0.158, and 0.819, respectively. $F$ was the most important hydrological parameter affecting the CS breeding population. Although the data on the broodstock population are not a consistent time series, temporal variation can provide a reliable reference for characterizing changes in CS habitat quality before and after the alteration of hydrological processes^7^.

Many previous studies have demonstrated that hydrological alterations threaten the spawning grounds and reproduction of migratory fish^13^. Inspired by IHAs, many studies always characterize hydrological alterations in regulated rivers by magnitude, frequency, timing, duration, and the change rate of flow^67^. The IHAs and flow duration curves are suitable for assessing or describing the natural flow regime of a river, but they cannot reflect the ecological implications of the river hydrological process and provide insight into river ecosystem protection and landscape restoration^68^. In the study by Li et al.^28^, the number, duration and growth rate of water rising processes were considered as characteristic flooding parameters. They explained that there is some regularity in the flow regimes during the spawning season. In the present study, the date and initial flow of the water rising process were added as additional parameters, and the five characteristic hydrological parameters ($N$, $\overline{T }$, $\overline{\eta }$, $D$, and $F$) were used to explicitly characterize the water rising process.

### Optimized ecological flow regime for fish spawning

Taking the Yichang section as an example, the five characteristic parameter values of the optimal daily ecological flow and the measured flow are shown in Table 3. It shows that the optimized ecological flow for FMCC spawning could improve $\overline{\eta }$ in wet and normal years, and decrease $F$ in the three hydrological years. Based on the ecological flow, from the mean value of characteristic parameters in three hydrological years, FMCC can obtain suitable breeding ground quality when the water rising process count and duration reach six times and 7.2 days, the daily flow increase rate will be above 2603 ${\mathrm{m}}^{3}/(\mathrm{s}\cdot \mathrm{d})$, the water rising process starts at the end of April with an initial flow of at least 7,227 m^3^/s in the Yichang section. Xu et al.^50^ and Li et al.^65^ analyzed the relationship between ecological hydrology index and natural spawning behavior of FMCC by correlation analysis. It was suggested that the suitable hydrological conditions for the reproduction of FMCC in the Yichang section should meet the following conditions: the water rising continues to rise for four days, and the daily average flow increases by more than 2000 ${\mathrm{m}}^{3}/(\mathrm{s}\cdot \mathrm{d})$. It indicates that the obtained optimum water rising process parameters are acceptable.

**Table 3:** Characteristic parameter values of measured flow and ecological flow in Yichang.

Time	Hydrological parameter	Wet year		Normal year		Dry year	
		Measured flow	Ecological flow	Measured flow	Ecological flow	Measured flow	Ecological flow
FMCC spawning period	$N$(times)	5	6	8	5	6	6
	$\overline{T }$(day)	8.60	7.5	6.50	7.0	4.00	7.2
	$\overline{\eta }$ $\left({\mathrm{m}}^{3}/(\mathrm{s}\cdot \mathrm{d})\right)$	2109	2742	1884	2932	4256	2136
	$D$(^th^ day)	144	122	112	115	142	118
	$F$(${\mathrm{m}}^{3}/\mathrm{s}$)	8800	8222	13,600	7299	9660	6161
CS spawning period	$N$(times)	2	2	4	3	2	3
	$\overline{T }$(day)	5.50	6.00	4.50	5.00	5.00	5.33
	$\overline{\eta }$ $\left({\mathrm{m}}^{3}/(\mathrm{s}\cdot \mathrm{d})\right)$	1392	1383	1158	1610	1035	967
	$D$(^th^ day)	275	281	298	274	297	275
	$F$(${\mathrm{m}}^{3}/\mathrm{s}$)	13,000	19,268	8850	17,900	9070	10,485

For CS spawning, the optimized ecological flows increase the duration of the water rising process and the initial flow in wet, normal, and dry years (Table 3). From the perspective of average characteristic parameters of ecological flow in three typical years, the ideal water rising process count and duration in Yichang should be three times and 5.4 days, the daily flow rising rate reached 1320 ${\mathrm{m}}^{3}/(\mathrm{s}\cdot \mathrm{d})$, the initial flooding occurred in early October, and the initial flow was more than 15,884 m^3^/s. Zhou et al.^60^ stated that the spawning ground flow in the middle reach of the Yangtze River should ideally be between 10,000 and 17,000 m^3^/s. The present study is close to the above results, and the proposed parameter values are all lower limits, which is more convenient to guide the reservoir operation.

The duration, rate, initial date, and flow of the water rising process obtained in this study are important indicators that affect fish reproduction^1,10^. The duration of the water rising reflects the persistence of the flood peak process. The longer the duration of the spillway peak, the larger the spawning scale^50^. In addition, inducing fish spawning requires a certain flow magnitude; the higher the initial flow, the shorter the spawning time^69^. The date and initial flow of the water rising process are two feasible time and magnitude parameters that can guide the timing of the ecological operation.

It is reported that the dramatic difference between the hydrological characteristics of the pre- and post-altered periods has long-term effects on aquatic life^24,68^. Many countries have been implementing ecological flows as a means of reducing hydrological alteration and mitigation of ecosystem degradation^67^. The restoration of the hydrological conditions can be achieved by maintaining the hydrological characteristic parameters at their pre-altered levels. The ecological flow optimization model proposed in this study provides the scientifically acceptable and cost-effective way to calculate ecological flow within different sites in inter-regional water resources planning^70^. In addition to the water rising process, water depth, flow velocity, and water temperature may also be important factors influencing native fish reproduction^60^. Therefore, the environmental quality assessment model should integrate hydrological factors and physical factors such as water temperature, water depth, and suspended sediment concentration on fish spawning in further studies. Moreover, releasing ecological flows in rivers may inevitably cause economic losses to upstream reservoirs. In further research, stakeholders should seek the benefits of a trade-off in conflicts between maintaining ecological flows and power generation.

## Conclusions

The study examined the changes in hydrological processes during the fish spawning season, as well as the ecological flow and eco-hydrological conditions essential for fish reproduction. The spawning activities of FMCC and CS in the middle reaches of the Yangtze River were taken as a case study. The following conclusions were drawn:

1. In the post-altered period, the water rising process duration, the daily flow increase rate, and the initial water rising flow at Jianli were significantly changed during the spawning period of FMCC. During the spawning period of CS, the water rising processes count and the initial water rising flow at Yichang were dramatically changed.
2. FMCC can obtain suitable spawning ground quality when the number and duration of water rising processes in the Yichang section reach six times and 7.2 days, the daily flow increase rate is above 2603 ${\mathrm{m}}^{3}/(\mathrm{s}\cdot \mathrm{d})$, the first flooding starts at the end of April with an initial water rising flow of at least 7227 ${\mathrm{m}}^{3}/\mathrm{s}$.
3. For the spawning activities of CS, the ideal number and duration of water rising processes in Yichang should be three times and 5.4 days, the daily flow increase rate should reach 1320 ${\mathrm{m}}^{3}/(\mathrm{s}\cdot \mathrm{d})$, and the first water rising occurred in early October with a flow of more than 15,884 ${\mathrm{m}}^{3}/\mathrm{s}$.

The proposed approach can be applied to other impounded rivers that require assessment of the variation in water rising processes and the creation of appropriate ecological flows for aquatic species.

## Supplementary Information

Supplementary Tables.

## Data Availability

The datasets generated during and/or analyzed during the current study are available from the corresponding author on reasonable request.
