# Supplementary material for: Identification and restoration of hydrological processes alteration during the fish spawning period
Source: Sci Rep. 2023 Jul 12;13:11307. doi: 10.1038/s41598-023-38441-x (PMC10338451; doi:10.1038/s41598-023-38441-x)
Supplement: Supplementary file 1 — Supplementary Tables. [file 41598_2023_38441_MOESM1_ESM.docx]

**Identification and restoration of hydrological processes alteration during the fish spawning period**

Journal name: Scientific Reports

Yang Yu^1^, Rui Zhao^1,^*, Jiahe Zhang^2^, Sen Du^1^, Tianyu Zhou^1^, Xingjia Fu^1^, Shuoyun Jiang^1^

^1^ Faculty of Geosciences and Environmental Engineering, Southwest Jiaotong University, No.999 Xi’an Road, Chengdu 611756, China

^2^ Sichuan Provincial Water Resources Department, River, and Lake Protection and Regulatory Affairs Center, No.33 Qingjiang Road, Chengdu 610072, China

* Corresponding author: Faculty of Geosciences and Environmental Engineering, Southwest Jiaotong University, No.999 Xi’an Road, Chengdu 611756, China

E-mail address: ruizhao@swjtu.edu.cn (RZ)

Telephone number: 86+15982814895

**Table A1.** General framework of NSGA-III-EO.

| Input: |
| --- |
| Multiobjective optimization problem; |
| a termination criterion; |
| $N$: the number of population (size) considered in NSGA-III-EO; |
| $\boldsymbol{Z}=\left\{ \boldsymbol{\lambda}^{1}, \boldsymbol{\lambda}^{2}, . . . ,\boldsymbol{\lambda}^{N} \right\}$: a set of $N$ reference points; |
| Output: |
| Approximation to the Pareto optimal set and the Pareto optimal objective vectors. |
| Step 1: Initialization: |
| Generate reference points ***Z***; |
| Generate an initial population $P_{0}$. Set $i=0$. |
| While the termination criterion is not met, generate a new offspring $Q_{i}$ from parents $P_{i}$ by applying binary crossover and polynomial mutation, and then combine parent and child populations, and set $R_{i}=P_{i}\cup Q_{i}$; |
| Nondominated sorting of $R_{i}$, set $\left( F_{1},F_{2},\cdots\right)$ which are all PF of $R_{i}$, and set $S_{i}=\emptyset, k=1$; |
| Step 2: Update: |
| Include all nondominated fronts in the new population $S_{i}$, set $k=k+1$ and $S_{i}=S_{i}\cup F_{k}$, until the size of new population is equal to or for the first time becomes larger than the size of the parent population, thus $\left\vert S_{i} \right\vert\geq N$, and the last front to be included is $F_{l}=F_{k}$; |
| Step 3: Termination criterion: |
| If $\left\vert S_{i} \right\vert=N$, then $P_{i+1}=S_{i},$break. Output $\boldsymbol{P}=\boldsymbol{PS}\cup\boldsymbol{PF}$; or else normalize the individuals in $S_{i}$ with reference points $\boldsymbol{Z}$, then eliminate individuals from $S_{i}$: $K=\left\vert S_{i} \right\vert-N$; |
| Associate the normalized individual in $S_{i}$ according to the perpendicular distance of each individual of $S_{t}$ from each of the $\boldsymbol{\lambda}^{i}$; |
| The worst individual whose associated reference point has the maximum niche count is eliminated from $S_{i}$, then $i=i+1$ and go to Step 2. |

**Table A2.** Classification of different hydrological years at Yichang and Jianli in the post-altered period.

| Section | Hydrological year | Flow range (m^3^/s) | Typical years |
| --- | --- | --- | --- |
| Yichang | Wet year | $Q\geq13184.3$ | 2000, 2005, 2012, 2014, 2017, 2018, 2019, 2020 |
|  | Normal year | $12825.7\leq Q<13184.3$ | 2001, 2003, 2004, 2008, 2016 |
|  | Dry year | $Q<12825.7$ | 2002, 2006, 2007, 2009, 2010, 2011, 2013, 2015 |
| Jianli | Wet year | $Q\geq12440.2$ | 2000, 2005, 2012, 2014, 2017, 2018, 2019, 2020 |
|  | Normal year | $11664.3\leq Q<12440.2$ | 2001, 2004, 2008, 2010, 2016 |
|  | Dry year | $Q<11664.3$ | 2002, 2003, 2006, 2007, 2009, 2011, 2013, 2015 |

**Table A3.** The fitting goodness indicators of structural equation model for effects of water-rising process on FMCCs.

| Fitting goodness indicator | Chi-square/ Degrees of freedom | RMSEA | CFI | GFI | IFI |
| --- | --- | --- | --- | --- | --- |
| Simulation results | 0.826 | 0.065 | 0.991 | 0.929 | 0.992 |
| Fitting criteria | $<3$ | $<0.08$ | $>0.90$ | $>0.90$ | $>0.90$ |

**Table A4.** The fitting goodness indicators of structural equation model for effects of water-rising process on CS.

| Fitting goodness indicator | Chi-square/ Degrees of freedom | RMSEA | CFI | GFI | IFI |
| --- | --- | --- | --- | --- | --- |
| Simulation results | 1.096 | 0.059 | 0.980 | 0.933 | 0.985 |
| Fitting criteria | $<3$ | $<0.08$ | $>0.90$ | $>0.90$ | $>0.90$ |

**Table A5.** Natural hydrological parameter values for Yichang and Jianli in pre-altered periods.

| Section | Hydrological year | During FMCC spawning period at Jianli (4.21-7.20) | | | | | | | | | | During CS spawning period at Yichang (10.1-11.30) | | | | | | | | | |
| --- | --- | --- | --- | --- | --- | --- | --- | --- | --- | --- | --- | --- | --- | --- | --- | --- | --- | --- | --- | --- | --- |
|  |  | $N_{0,mean}^{T_{a}}$ | $N_{0,max}^{T_{a}}$ | $\bar{T}_{0,mean}^{T_{a}}$ (d) | $\bar{T}_{0,max}^{T_{a}}$ (d) | $\bar{\eta}_{0,mean}^{T_{a}}$  $\left( m^{3}/(s\cdot d) \right)$ | $\bar{\eta}_{0,max}^{T_{a}}$  $\left( m^{3}/(s\cdot d) \right)$ | $D_{0,mean}^{T_{a}}$ | $D_{0,max}^{T_{a}}$ | $F_{0,mean}^{T_{a}}$ | $F_{0,max}^{T_{a}}$ | $N_{0,mean}^{T_{b}}$ | $N_{0,max}^{T_{b}}$ | $\bar{T}_{0,mean}^{T_{b}}$  (d) | $\bar{T}_{0,max}^{T_{b}}$  (d) | $\bar{\eta}_{0,mean}^{T_{b}}$  $\left( m^{3}/(s\cdot d) \right)$ | $\bar{\eta}_{0,max}^{T_{b}}$  $\left( m^{3}/(s\cdot d) \right)$ | $D_{0,mean}^{T_{b}}$ | $D_{0,max}^{T_{b}}$ | $F_{0,mean}^{T_{b}}$ | $F_{0,max}^{T_{b}}$ |
| Yichang | Wet | 5 | 9 | 8 | 14 | 2776 | 3709 | 131 | 169 | 8267 | 14400 | 2 | 6 | 6 | 13 | 1382 | 3567 | 281 | 300 | 19268 | 27600 |
|  | Normal | 5 | 7 | 7 | 12 | 2885 | 5809 | 131 | 172 | 7352 | 11200 | 3 | 5 | 5 | 9 | 1610 | 5425 | 279 | 293 | 18542 | 23800 |
|  | Dry | 6 | 9 | 7 | 10 | 2126 | 3916 | 124 | 138 | 6411 | 8600 | 2 | 4 | 5 | 8 | 1184 | 4205 | 283 | 309 | 15547 | 25200 |
| Jianli | Wet | 6 | 7 | 7 | 10 | 1736 | 2242 | 120 | 129 | 6449 | 9350 | 2 | 5 | 6 | 8 | 763 | 1449 | 283 | 290 | 14286 | 15700 |
|  | Normal | 5 | 7 | 7 | 8 | 1869 | 2365 | 126 | 146 | 6007 | 7340 | 2 | 3 | 5 | 9 | 1409 | 3950 | 290 | 311 | 13570 | 18100 |
|  | Dry | 4 | 5 | 9 | 10 | 1605 | 2345 | 132 | 143 | 7529 | 8470 | 3 | 4 | 5 | 7 | 766 | 1374 | 279 | 292 | 15971 | 19500 |
